# Enhanced Experimental Setup and Methodology for the Investigation of Corrosion Fatigue in Metallic Biodegradable Implant Materials

**DOI:** 10.3390/ma17215146

**Published:** 2024-10-22

**Authors:** Lukas Schumacher, Ikra-Nur Cetin, Sira Bielefeldt, Frank Rupp, Ariadne Roehler

**Affiliations:** Department of Medical Materials Science and Technology, Institute of Biomedical Engineering, University Hospital Tuebingen, 72076 Tübingen, Germanyfrank.rupp@med.uni-tuebingen.de (F.R.); ariadne.roehler@med.uni-tuebingen.de (A.R.)

**Keywords:** corrosion fatigue, three-point bending test, zinc, experimental setup, open-circuit potential, potentiostatic polarization, biodegradable implants

## Abstract

Biodegradable implants as bone fixations may present a safe alternative to traditional permanent implants, reducing the risk of infections, promoting bone healing, and eliminating the need for removal surgeries. Structural integrity is an important consideration when choosing an implant material. As a biodegradable implant is being resorbed, until the natural bone has regrown, the implant material needs to provide mechanical stability. However, the corrosive environment of the human body may affect the fatigue life of the material. Conversely, mechanical stress can have an effect on electrochemical corrosion processes. This is known as corrosion fatigue. In the presented work, an experimental setup and methodology was established to analyze the corrosion fatigue of experimental bioresorbable materials while simultaneously monitoring the electrochemical processes. A double-walled measurement cell was constructed for a three-point bending test in Dulbecco‘s Phosphate-Buffered Saline (DPBS^− −^), which was used as simulated body fluid (SBF), at 37 ± 1 °C. The setup was combined with a three-electrode setup for corrosion measurements. Rod-shaped zinc samples were used to validate the setup’s functionality. Preliminary static and dynamic bending tests were carried out as per the outlined methodology to determine the test parameters. Open-circuit as well as potentiostatic polarization measurements were performed with and without mechanical loading. For the control, fatigue tests were performed in an air environment. The tested zinc samples were inspected via scanning electron microscopy (SEM). Based on the measured mechanical and electrochemical values as well as the SEM images, the effects of the different environments were investigated, and the setup’s functionality was verified. An analysis of the data showed that a comprehensive investigation of corrosion fatigue characteristics is feasible with the outlined approach. Therefore, this novel methodology shows great potential for furthering our understanding of the effects of corrosion on the fatigue of biodegradable implant materials.

## 1. Introduction

One of the foremost causes of implant failure in orthopedic applications is implant-associated infection, which can lead to corrective surgeries, causing additional costs as well as considerable patient discomfort [1,2]. Infections are among the most common and severe complications linked with biomedical devices [3]. A total of 25.6% infections in US health care stem from medical device-associated infections [3,4]. Biodegradable bone fixation implants may be a safe alternative to traditional implants. Biodegradable implants might lower the risk of infections by making the need for a second surgery redundant as well as by having antibacterial properties and promoting the natural healing of bone tissue [1,2].

Biomechanical implants of any type need to fulfill specific requirements. They need to be both biologically compatible as well as able to properly replace or enhance functionality [5]. The ongoing development of and research on materials are of great importance to consistently improve treatment results. Biodegradable materials can be used as bone fixations or scaffolds to treat bone defects. They provide necessary mechanical stability while degrading inside the patient’s body as the bone tissue regrows [6,7,8]. Consequently, they can reduce the risk of complications often associated with permanent implants, such as chronic inflammation, physical irritation, and infections [6,7,8,9,10,11]. During the process of degradation, to prevent implant failure, the implant needs to maintain mechanical integrity until the bone has sufficiently regrown. Ideally, the rate of degradation is equal to the natural bone growth rate, being fully degraded by the time the bone has completely regrown [6,8,12]. Currently, implants made of zinc, magnesium, iron, and their alloys are being investigated for their suitability as biodegradable implant materials [5,7,8,10,12,13,14,15,16,17,18,19].

In order to investigate the biodegradation characteristics of metals, different basic research methods are applied. The corrosion rate of a metal seems to be a valid indicator in predicting the time needed to degrade inside the human body [20]. Besides degradation, mechanical properties, specifically the fatigue life, also play a decisive role. The fatigue failure of a material is described as the delayed onset of fracture resulting from cracks caused by cyclic mechanical loading with stresses well below the material’s maximum mechanical strength [21]. The synergy of mechanical fatigue and electrochemical processes caused by a corrosive medium is called corrosion fatigue [21], and it is an important consideration for load-bearing implants.

There are testing methods for investigating the corrosion fatigue behavior of materials by performing bending tests on standardized samples. The results are typically represented in graphs of the number of cycles until failure as a function of the applied stress amplitude. Measurements in aqueous environments can then be compared to measurements in an air environment to draw conclusions about the impact of a certain corrosive environment on the fatigue life of a material [21,22].

Some studies reported the simultaneous investigation of mechanical and electrochemical behavior. Gu et al. [23] conducted an experiment to investigate the corrosion fatigue of a magnesium coating under cyclic loading with a focus on its relation to implant failure. Samples under mechanical loading showed a higher corrosion rate compared to those without mechanical loading. Grell et al. [24] conducted three-point bending tests of glassy Zr-based alloys under electrochemical monitoring in a sodium sulfate–saline solution. The results revealed the formation of cracks at corrosion pits. Such corrosion pits were generated at localized breakthrough points of the sample’s passive layer. Li et al. [6] carried out experiments on porous zinc to investigate its fatigue properties in both an ambient atmosphere and a simulated body fluid (SBF) at 37 °C using an electro-dynamic mechanical testing machine. The results show that the fatigue limit was higher in the SBF than in air, and the majority of corrosion products accumulated around the punch mark area. Jafari et al. [25] conducted static tests as well as cyclic fatigue tests on a magnesium alloy in a modified SBF at 37 °C as well as in air and demonstrated that the magnesium samples alloyed with zinc exhibited higher corrosion resistance.

In order to advance the development of biodegradable implants and, specifically, to make more accurate predictions about the mechanical integrity over the course of the degradation process, it is necessary to combine mechanical testing with electrochemical monitoring. The aim of this study is to establish a setup that allows for comparative fatigue testing of rod-shaped metallic samples in air as well as in different media while utilizing electrochemical analytical methods to simultaneously monitor corrosion processes. The combination of corrosion fatigue testing and different electrochemical methods may help create a more fundamental understanding of corrosion processes. The experimental framework employed by Grell et al. [24] served as a basis. Initial experiments and configurations were conducted to investigate the mechanical and electrochemical properties, respectively. In addition, a methodology is outlined for determining the appropriate testing parameters for the corrosion fatigue testing of sample materials of different mechanical properties. Following this, the established methodology and setup underwent validation with zinc.

## 2. Materials and Methods

Zinc was chosen as an exemplary sample material for this study. Additionally, this metal is regarded as a promising material for biodegradable implants [6,8,13,14,26]. The work was subdivided into different stages. Preliminary static bending tests were conducted to find a reference point for the sample properties and parameters to be used in later tests. Here, values from similar experimental setups in the literature served as a basis [27]. Following this, a preliminary dynamic parameter study was conducted with the aim of determining suitable parameters for a dynamic fatigue test. Based on the obtained parameters, dynamic three-point bending tests were performed in air, serving as a control group. Furthermore, a three-electrode cell was configured. The open-circuit potential (OCP) of the Zn samples was monitored, and potentiostatic polarization (PSP) measurements were performed in Dulbecco′s Phosphate-Buffered Saline (Thermo Fisher Scientific Inc., Waltham, MA, USA) without CaCl_2_ and MgCl_2_ (DPBS^− −^, see Table 1), which was used as an SBF. Finally, dynamic three-point bending tests in the SBF were carried out while monitoring the OCP and during polarization.

**Figure 1:**
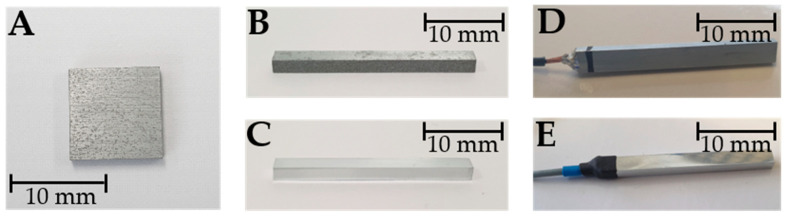
(**A**) Rough square zinc sample, 10 × 10 × 2 mm^3^; (**B**) rough zinc bending test sample, 27 × 3 × 2 mm^3^; (**C**) polished bending test sample after grinding; (**D**) electrical contacting with copper wire; (**E**) heat shrink tubing as insulation for soldering joint (blue: 0.8 mm and black: 1.6 mm).

For this work, zinc samples (HMW Hauner GmbH & Co. KG, Röttenbach, Germany; purity 99.99%) of two geometries were used: square samples measuring 10 mm × 10 mm × 2 mm (Figure 1A) and rod-shaped bending test samples of 27 mm × 3 mm × 2 mm (Figure 1B).

### 2.1. Preparation of Zinc Samples

The sample surfaces were prepared in three different steps. First, the zinc samples were successively ground with SiC P1200, P2500, and P4000 paper (Buehler, ITW Test & Measurement GmbH, Leinfelden-Echterdingen, Germany) at 150 rpm using a grinding and polishing machine with a platen 305 mm in diameter (Buehler Metaserv Motopol 12, ITW Test & Measurement GmbH, Leinfelden-Echterdingen, Germany).

After grinding (Figure 1C), all samples were rinsed with acetone, isopropanol, and deionized water and dried with purified compressed air. Finally, the samples were electrically connected by soldering copper wires to the ends of the samples (Figure 1D). The soldering joints were isolated using heat shrink tubing (Figure 1E) and again cleaned as described above.

### 2.2. Preliminary Mechanical Tests

The mechanical preliminary tests in a liquid medium were categorized into static preliminary tests and a dynamic parameter study. The static tests served to determine the general mechanical strength of the samples as a basis for the dynamic testing. The dynamic tests then determined a suitable loading force for the chosen number of cycles in the corrosion fatigue experiments. Dynamic tests intend to cause high cycle fatigue, where fatigue typically occurs at lower (elastic) stress values, well below the material’s yield strength, with a high number of cycles, whereas low cycle fatigue stems from repeated plastic deformation [28,29].

The outlined approach is intended to work for a variety of implant materials.

#### 2.2.1. Static Three-Point Bending Test

First, static three-point bending tests were conducted using a universal testing machine (Z010, ZwickRoell GmbH & Co. KG, Ulm, Germany), with loading and support pin radii of 1.5 mm and a support span of 20 mm. The entire setup was immersed in a bath of deionized water and heated to 37 ± 1 °C. The initial force value was set to 1 N, with a testing speed of 0.5 mm/min. Five Zn samples were tested. Due to the ductility of zinc, no fracturing of the samples was reached, and the measurements were terminated at a deflection of approximately 10 mm. Samples of less ductile materials can be loaded until failure due to fracturing. The maximum force measured in the elastic area of the stress–strain curve should be used as a point of reference for the subsequent dynamic bending test.

#### 2.2.2. Dynamic Parameter Study

**Figure 2:**
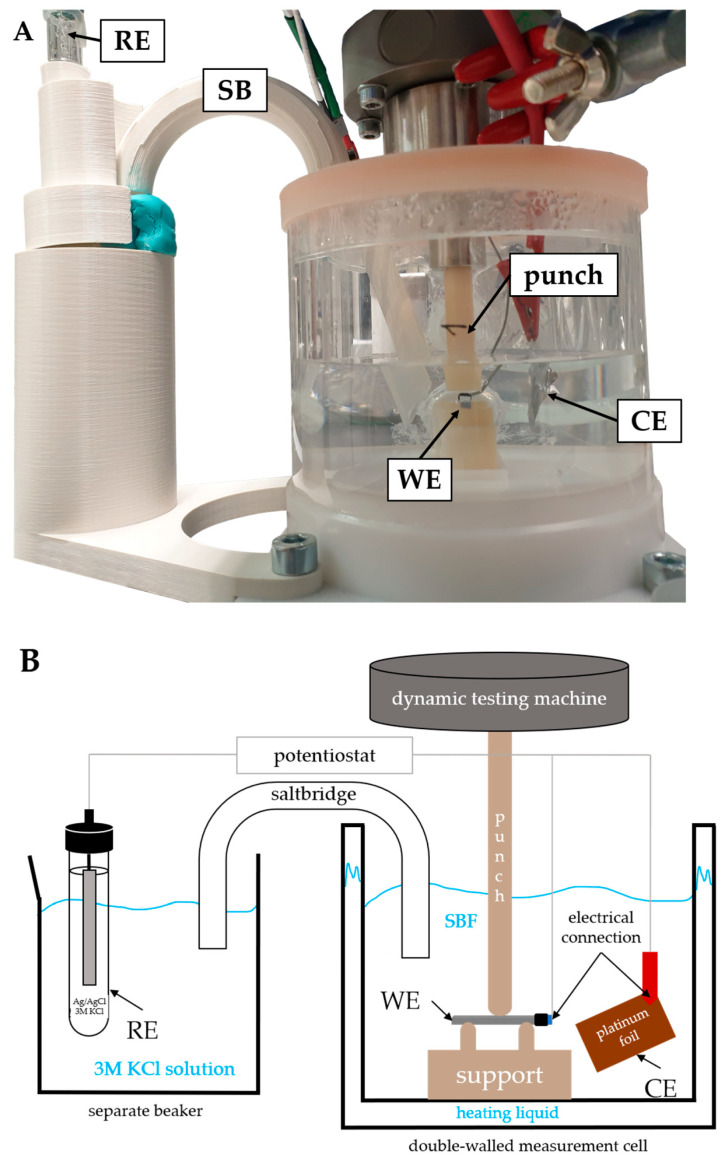
The experimental setup for investigating corrosion fatigue: (**A**) a photograph with labeling; (**B**) a schematic representation with labeling. A double-walled measurement cell mounted to the base plate of the dynamic testing machine. The working electrode (WE) and counter electrode (CE) are placed in the measurement cell filled with simulated body fluid (SBF) at 37 °C. The reference electrode (RE) is placed in a separate beaker filled with a KCl solution and connected by a salt bridge (SB) which is held in an additively manufactured holder.

The dynamic three-point bending parameter study was performed using a force-controlled testing machine (KN5, DYNA-MESS Prüfsysteme GmbH, Aachen, Germany). The support span, support pin radius, and sample geometry were the same as those used for static testing. The support and punch were milled from zirconium dioxide (priti multidisc ZrO_2_ High Translucent, pritidenta GmbH, Leinfelden-Echterdingen, Germany). The setup was immersed in 300 mL of deionized water, which was heated to 37 ± 1 °C using a circulation thermostat inside a double-walled measurement cell (Figure 2). Eight samples were prepared by grinding and cleaning.

During the experiment, the samples were subjected to cyclic loading at a frequency of 15 Hz until one of two termination criteria was met: (1) sample failure due to fracture or reaching the predefined maximum deflection of 1 mm or (2) reaching of the predefined maximum number of 5 × 10^5^ loading cycles. The basis for the sequence of measurements was the standard DIN 50100 for load-controlled fatigue testing [22]. If criterion (1) was met for a given sample, the maximum force was reduced for the following measurement. If criterion (2) was met, the force was increased for the subsequent measurement. The initial maximum force was set to 20 N, as this was determined to be the upper limit of the elastic range in the static bending tests. The following seven samples were loaded with the following maximum forces: 20 N, 15 N, 10 N, 5 N, 8.25 N, 10 N, 8.25 N, and 5 N. Force, deflection, and the number of cycles were recorded for each specimen. The relative deviation from the nominal force was then calculated from the recorded data of the testing machine, i.e., via the actual applied forces.

### 2.3. Preliminary Electrochemical Tests

These experiments served to determine the effect of the resistance added by a salt bridge (SB) on the measured potential and current density, as it is important to select an SB which has an adequately small resistance. For all electrochemical experiments, a three-electrode setup was used. For the preliminary tests, this setup consisted of the prepared square-shaped Zn samples as working electrodes (WEs), an Ag/AgCl reference electrode (RE) (Metrohm, Herisau, Switzerland), and a platinum counter electrode (CE) (Metrohm, surface area: 112 mm^2^) in SBF as medium. The experiments were carried out using a potentiostat (VersaSTAT 3, AMETEK Scientific Instruments, Oak Ridge, TN, USA).

**Figure 3:**
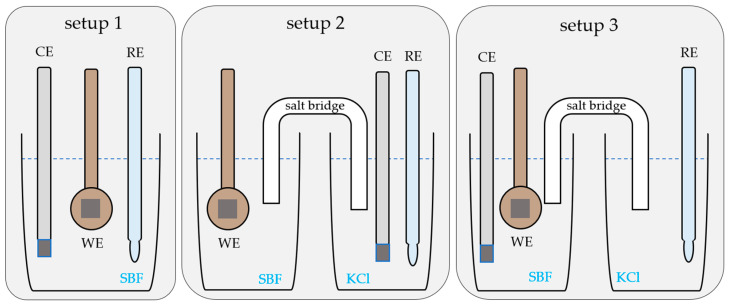
Experimental setups 1, 2, and 3 using the three-electrode cell configuration consisting of an RE, WE, and CE. Setup 1 shows all electrodes in one cell, and setups 2 and 3 show the electrodes placed in two separate beakers connected by a KCl salt bridge. The Zn sample serves as the WE.

Due to spatial constraints, the RE was not placed in the measurement cell below the testing machine with the WE and CE, as shown in Figure 3, setup 1. Instead, two alternative setups were designed, where either the RE (Figure 3, setup 3) or both the RE and CE (Figure 3, setup 2) were placed in a separate beaker containing a 3 M KCl solution and connected to the cell using an SB. Two kinds of SBs with internal diameters of 8 mm (SB+) and 4 mm (SB−), respectively, were prepared by mixing a 3 M KCl solution (KCl, ≥99.5%, p.a., ACS, ISO, Carl Roth GmbH + Co. KG, Karlsruhe, Germany) with 2 vol% agar powder (microbiology tested, Sigma-Aldrich Chemie GmbH, Steinheim, Germany). The solution was solidified in a silicone tube. Between measurements, the SBs were stored at 7 °C, with the ends immersed in 3 M KCl.

OCPs of square-shaped samples were measured at a temperature of 37 ± 1 °C. Furthermore, the samples were polarized potentiostatically at −0.7 V_Ag/AgCl_ over a duration of 30 min.

### 2.4. Corrosion Fatigue

To investigate the corrosion fatigue characteristics, a dynamic three-point bending test setup was combined with a three-electrode configuration to monitor electrochemical processes. Additionally, separate control experiments were conducted to assess the mechanical and electrochemical aspects separately.

In order to evaluate the effect of an aqueous, corrosive environment on the fatigue properties of a sample, a dynamic three-point bending test was additionally conducted in a dry environment. Three experiments were carried out in a non-climate-controlled environment (20–25 °C) with a frequency of 15 Hz, a cycle number of 5 × 10^5^, and a maximum force of 8.25 N. As a second control group, OCP and PSP measurements were performed in SBF without mechanical loading using Setup 3 (Figure 3). For OCP measurements, 240 mL of SBF (37 °C), a separate beaker containing 60 mL of 3 M KCl, and an SB ($\emptyset_{inner}$ = 8 mm) were used. For the PSP measurements, 300 mL of SBF (37 °C) was used. The samples were polarized at −0.95 V_Ag/AgCl_ for 9.25 h, corresponding to the maximum number of cycles in the mechanical control group. The potential of −0.95 V_Ag/AgCl_ was determined by performing potentiodynamic polarization measurements, during which a weak pseudo-passivity was observed in a range between approx. −1.05 and −0.85 V_Ag/AgCl_. For the static polarization, the midpoint of this range was chosen.

Finally, for the investigation of the corrosion fatigue, the setups from the control experiments were combined (Figure 2) using the configuration of setup 3 (Figure 3). Due to limited space, a platinum foil (VENTRON—GmbH, Karlsruhe, Germany; surface area: 1250 mm^2^) was used as the CE. The temperature-controlled (37 °C) measurement cell was filled with 240 mL of SBF for OCP measurements and 350 mL for PSP, respectively. The sample was held in place by 3D-printed clamps. A separate beaker for the RE was filled with 50 mL of KCl. The OCP was continuously measured, and the mechanical loading process was started after the stabilization of the potential. For the OCP measurements with mechanical loading (M-OCP), the OCP measurement was continued during mechanical loading for 9.25 h. For the PSP group with mechanical loading (M-PSP), the sample was polarized at −0.95 V_Ag/AgCl_ during loading. Three measurements were performed for each group with the maximum force of the mechanical loading set to 8.25 N.

To remove corrosion products after the experiments, the samples were cleaned using a soft polymer brush and rinsed with deionized water. Subsequently, they were cleaned—based on DIN EN ISO 8407 [30]—in 10 mL of a glycine solution (231.5 g/L) in an ultrasonic bath at 25 °C for 15 min and dried. This method is suitable for the removal of zinc corrosion products. For other materials, different methods may need to be applied. Each sample surface was inspected using a scanning electron microscope (SEM) LEO 1430 (Carl Zeiss AG, Oberkochen, Germany).

## 3. Results and Discussion

The experiments revealed design aspects and parameters that are of great importance for the high repeatability and accuracy of the corrosion fatigue measurements. It is crucial to handle sample preparation with great care to avoid damaging the surface, which could influence electrochemical measurements. For the connection of the samples, flexible copper wire as well as insulation should be used in order to achieve precise and secure positioning on the support pins.

### 3.1. Mechanical Preliminary Tests

#### 3.1.1. Static Three-Point Bending Test

**Figure 4:**
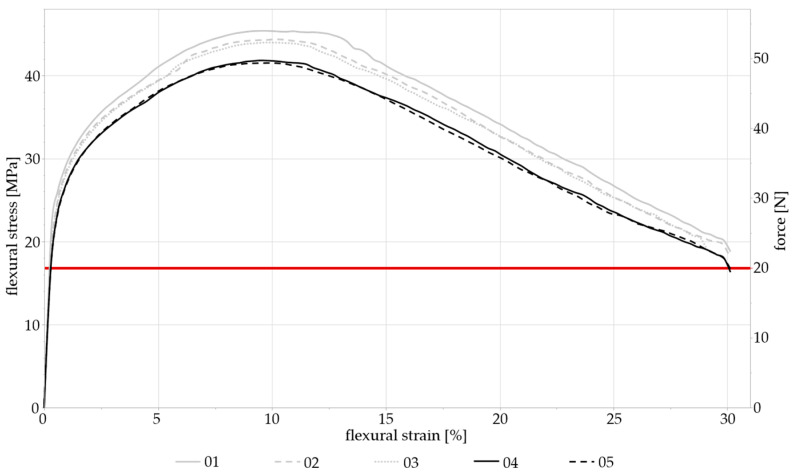
The flexural stress–flexural strain curve of the static three-point bending test for all five specimens. The force recorded by the machine is indicated on the second y-axis. The end of the linear area is marked in red at ~20 N.

Due to the ductility of zinc, the samples did not fracture during static three-point loading, but they were initially plastically deformed before being pushed downwards from the supports. In the bending test, the samples were deformed continuously (see Figure 4). The mean maximum flexural stress attained was 43.16 MPa ± 1.64 MPa, with the end of the linear area of the resulting stress–strain curve being at ~20 N. This value was then chosen as the starting point for the subsequent dynamic loading measurements in order for the dynamically applied forces to remain in the elastic region.

#### 3.1.2. Dynamic Parameter Study

**Figure 5:**
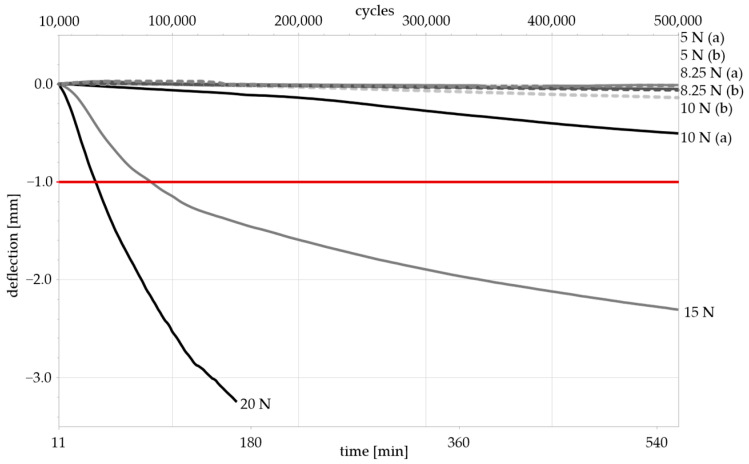
A normalized deflection cycle diagram of dynamic loading. The failure limit for criterion (1) is marked in red at −1 mm. The measured maximum load amplitudes were 5 N (2 samples with (a) and (b)), 8.25 N (2 samples), 10 N (2 samples), 15 N (1 sample), and 20 N (1 sample).

Figure 5 shows the deflection of the specimen against the number of load cycles and time. The deflection is normalized to the punch position after the transient process of the machine. A measurement exceeding a deflection of −1 mm was considered “failed” due to meeting termination criterion (1) (see Section 2.2.2).

None of the tested specimens fractured. The ones loaded with 20 N and 15 N, however, were deformed so severely that they met termination criterion (1), exceeding a deflection of 1 mm. The samples loaded with 10 N, 8.25 N, and 5 N all met criterion (2) (see Section 2.2.2). The samples loaded with 5 N showed the lowest deformation (see Table 2).

However, at 8.25 N, the relative deviation between the set force of the testing machine and the actual applied force was the lowest (Table 3). Therefore, 8.25 N was deemed the most accurate and selected for the subsequent experiments while remaining in the elastic region, as determined in Section 3.1.1. The accuracy of the available testing equipment should be considered when selecting the testing parameters. For the investigation of materials with a low elastic limit, machines that are capable of accurately measuring small forces should be used. Alternatively, the sample geometry could be modified, albeit with due regard to a potential loss of comparability.

### 3.2. Electrochemical Preliminary Tests

**Figure 6:**
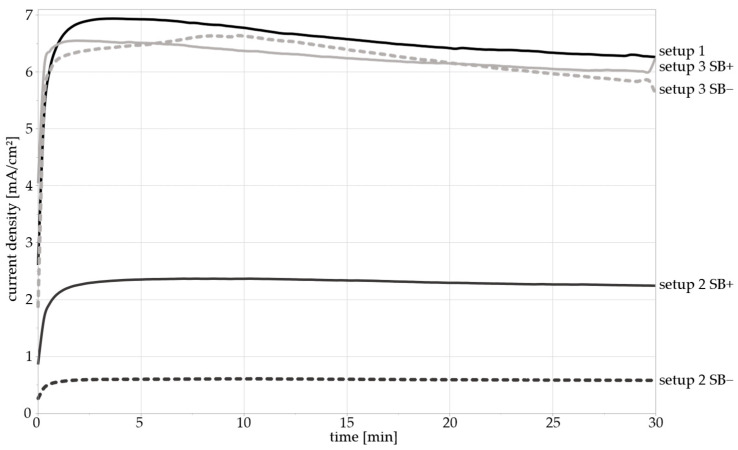
Comparison of setups 1–3 and SB thickness: Current density–time diagram of PSP measurements with 8 mm (SB+) and 4 mm (SB−) salt bridges in setups 2 and 3. Setup 1 did not include SB. WE was polarized at 0.7 V_Ag/AgCl_.

Preliminary investigations were carried out to analyze the impact of using an SB as part of the setup, its diameter, as well as of the electrode placement. The results of the OCP measurements revealed no significant difference between the three different electrode configurations. All potentials stabilized in a range between −1.05 and −1 V_Ag/AgCl_. However, the results demonstrate that the diameter of the SB affects the measured current densities severely in setup 2, i.e., with the CE and RE in the separate beaker. Figure 6 shows the current density of the PSP measurements over time for setups 1–3, as well as for the two SB diameters. The curves for setups 1 and 3 are very similar, regardless of the diameter used, with current densities between 5.9 and 6.7 mA/cm^2^. In contrast, setup 2 shows significantly lower current densities.

The results also show a lower current density with the small SB diameter compared to the larger diameter, ranging from 2.1 to 2.2 and from 0.51 to 0.59 mA/cm^2^, respectively. This is likely due to their smaller cross-sectional area causing a higher electrical resistance in the circuit and, therefore, a lower conductance, leading to decreased current densities, in accordance with the formula for resistance:

R=ϱ·l/A

where ϱ is the resistivity, l is the length, and A is the cross-sectional area of the conductor [31].

In setup 3, where only the RE was placed in a separate beaker, the current densities during polarization were sufficiently close to the values measured using setup 1. The measured potentials were unaffected by the different setups. Setup 3 with a large-diameter SB was therefore the preferred configuration and selected for the corrosion fatigue experiments.

The amount of SBF used in the experiments with rod-shaped samples was higher than that with square-shaped samples in order to achieve a similar electrolyte-to-sample surface area ratio. A similar consideration applies to the CE, which, according to the literature, should have a significantly larger surface area than the working electrode [32]. In the preliminary tests, the commercial electrode was used, whereas for the rod-shaped samples, a platinum foil was used, whose surface area was 11 times as large.

A limitation of this study was that the electrochemical experiments without loading were conducted in a heated beaker rather than the measurement cell. For future experiments, it is recommended to perform all electrochemical measurements within the three-point bending configuration, even if no loading is applied, to improve result comparability.

### 3.3. Corrosion Fatigue

The double-walled temperature-controlled measurement cell made from polymer and equipped with zirconia-based supports and punch has been shown in this study to be suitable for mechanical fatigue testing with simultaneous electrochemical monitoring of corrosive processes. To optimize sample handling and ensure result reproducibility, some adjustments need to be made: During the experiments, some evaporation of the aqueous medium was noticed. Therefore, it would be advantageous for future experiments to add a lid to the measurement cell with an opening specifically for the loading pin. Furthermore, it was observed during the preliminary experiments that, on some occasions, the samples moved and slid laterally from the support pins during mechanical loading. To counteract this, 3D-printed clamps were used. For future experiments, the samples could be secured by modifying the pin design with barriers on the sides. This will simplify sample handling during the setup. Additionally, the shrink tubing might be replaced with a more flexible sealing compound [33] or insulating lacquer to shield the electrical contacts from the medium as the stiffness of the tubing contributed to the shifting of the sample. The wire and insulation should be as flexible as possible.

#### 3.3.1. Mechanical Evaluation of Results

**Figure 7:**
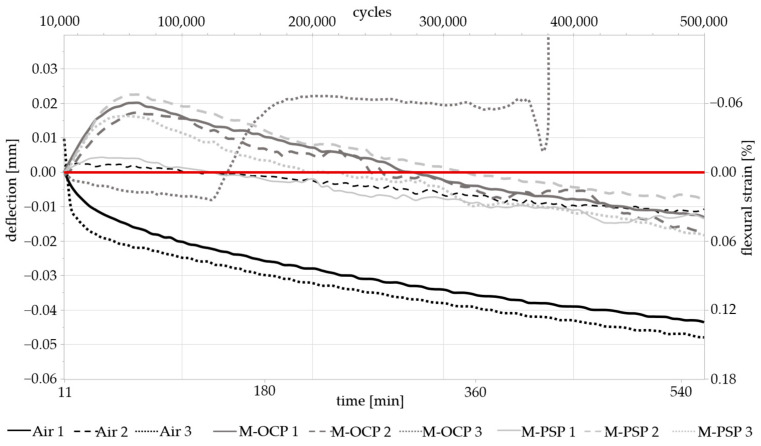
Comparison of deflection under mechanical stress in air and liquid (SBF): three control measurements in air (Air 1–3) and six measurements in liquid medium (M-OCP 1–3 and M-PSP 1-3). Loading force was set to 8.25 N, and loading duration was approx. 9.25 h. The red line marks a deflection of 0.00 mm.

The results of the mechanical measurements are presented in Figure 7, showing the deflection and strain against the number of cycles and time. The deflection was normalized to the punch position after the machine’s settling period of ~10,000 cycles. The deflection is indicated by a minus sign since the punch moves downwards along the y-axis. The experiments compared the effect of mechanical stress on the samples in air as well as in a liquid medium.

While none of the samples fractured during testing, material fatigue over the course of the 500,000 loading cycles was observed in all groups. The Air-1 and -3 samples showed the strongest fatigue (i.e., deflection relative to “0”), while the relative deflection was smaller for samples in a liquid medium, coinciding with the results presented by Li et al. [6]. However, this may also suggest that the chosen cycle number was simply not large enough for the medium to have any significant impact on the fatigue life of the material. In contrast to the measurements taken in air, most measurements in the SBF with electrochemical monitoring initially showed a deflection in the positive direction, i.e., an “upward” displacement. To determine the exact cause for this, further experiments need to be conducted. A likely explanation could be the sample momentarily losing contact with the support pins, due to buoyancy in the SBF, following contact with the loading pin. This may cause the loading pin to touch the sample and record a force value before the sample has settled back down on the supports. For better reproducibility and easier sample handling, modifications to the support pins are suggested, as the movement of the sample on the supports represents a main drawback of the presented experimental setup. In future iterations of the design, the prevention of sample movement—without interfering with mechanical testing—is expected to result in more accurate measurements.

For the zinc samples, the selected duration of 500,000 cycles was too short to observe any clear enhancement in fatigue phenomena by corrosion processes. Even though the M-PSP samples exhibited a more noticeably corroded surface in an SEM inspection, the accelerated corrosion reactions caused by the potentiostatic polarization had no discernable effect on the material fatigue over the measured period of time. No cracks could be observed on any of the tested samples in a visual (SEM) inspection. Figure 5 shows that during the preliminary measurements, one of the samples at 10 N deformed strongly, indicating that the selected loading force may have been too high, even though it was still well below the theoretical limit of elasticity. Consequently, the testing protocol should be adapted to a lower force value when using a higher number of cycles to examine fatigue properties more accurately. The number of cycles required for the occurrence of material fatigue highly depends on the properties of the material. Therefore, an initial dynamic measurement with an unlimited number of cycles (until fatigue failure) is recommended. For the experiments presented by Li et al. [6], up to 5 × 10^6^ cycles were reached, albeit different experimental setup, loading type, and sample geometry were utilized. However, it has to be considered that one important drawback of extending the experiment duration might be the loss of liquid medium due to evaporation over the course of a multi-day measurement, which may cause differences in the corrosion processes. A system to accurately compensate for liquid volume loss would need to be implemented. Longer experiments can also be conducted to investigate the impact of imposed potentials on the material fatigue. In future experiments, different corrosive media could be used as an alternative to sample polarization while monitoring the OCP.

An effective method for investigating the impact of corrosion processes on the fatigue behavior of a material could be conducting the presented experiments on samples that were stored in a corrosive environment for predefined durations prior to the measurements. The electrochemical processes can be monitored during mechanical loading.

#### 3.3.2. Electrochemical Evaluation of Results

**Figure 8:**
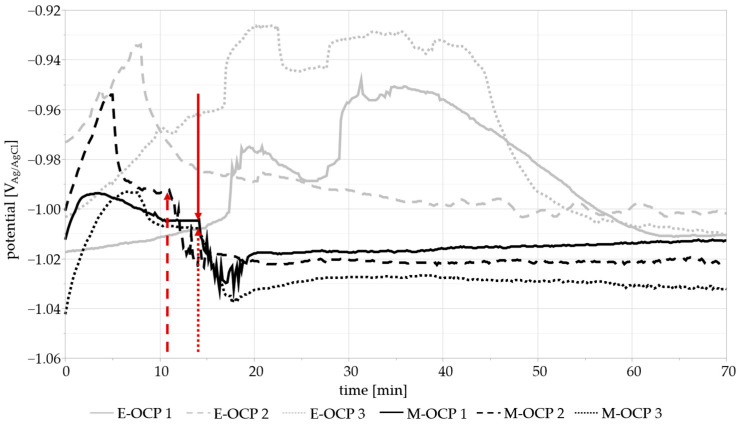
Comparisons of the first 70 min of the OCPs of the purely electrochemical measurements (E-OCP 1–3) and mechanically loaded samples (M-OCP 1–3). The start of mechanical testing is marked by the respective arrows.

OCPs and PSPs with mechanical load (M-OCP and M-PSP) were compared to the measurements without load (E-OCP and E-PSP). Figure 8 shows the first 70 min of the 9.25 h measurement. The start of mechanical loading is indicated by the respective red arrows. Mechanical loading was started after the initial stabilization of the potential (approx. 10–20 min) between −1.015 V and −0.999 V. At the beginning of loading, a fluctuation in the potential could be observed for ~5 min before stabilization at slightly more negative (cathodic) potentials of −1.04 V and −1.02 V, when the testing machine reached a stable frequency of 15 Hz. This may have been due to the formation of a layer of corrosion products on the sample surface when there was no mechanical loading. This film could cause a weak pseudo-passive effect, preventing, to some degree, the metal from reacting with the solution. However, the continuous motion of the loading pin may cause the layer to detach from the surface and disperse throughout the liquid medium, similar to what was reported by Grell et al. [24].

The samples without mechanical loading stabilized at −1.02 V and −0.99 V after approx. 50–70 min, much later than the M-OCPs. This may be attributed to the sample positioning during the measurement, which was conducted in a beaker and not in the measurement cell. Due to this positioning, it is possible that it took a longer time for the sample to reach an equilibrium between the reactions. The sample was not fixed as firmly as the M-OCP samples and could move slightly within the medium, possibly prolonging the formation of a film on the sample surface.

**Figure 9:**
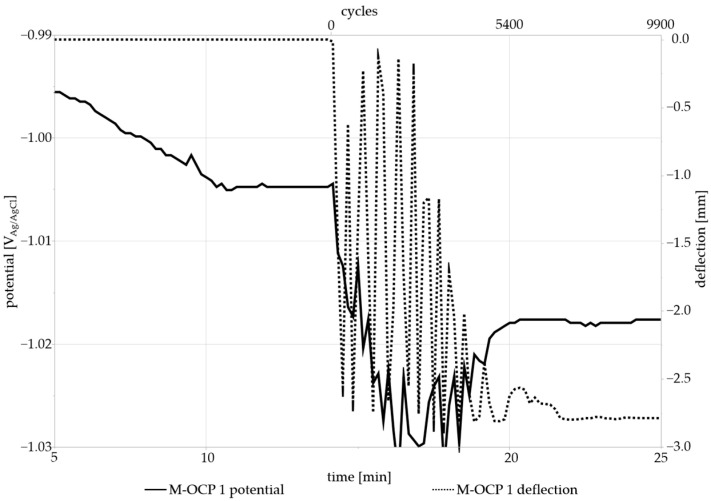
Minutes 5 to 25 of the M-OCP1 measurement showing the mechanical settling process. Once the OCP has stabilized for the first time, the mechanical stress starts at approx. 14 min.

In order to examine the fluctuation in the potential in more detail, Figure 9 includes the mechanical settling process of the testing machine in addition to the selected M-OCP 1 curve. The deflection is indicated on a second y-axis, and the elapsed time and cycles are indicated on the x-axis. It can be observed that the settling process affects the measured OCP. Each downward and upward movement of the punch causes the potential to rise and fall. The cause of the potential change corresponding to punch movement could be due to the disturbance of the sample surface by the punch. As the punch is pushing down on the sample, the film of corrosion products as well as the surface of the sample itself are damaged. The metal underneath is exposed, and its reaction with the medium may result in changes in the measured electrochemical behavior. The same is true for the support pins on which the sample is placed. After the settling process is completed, the sample OCP stabilizes at a more negative value. The monitoring of the OCP may give information about the influence of movement and dynamic stress on the corrosion potential of a sample during immersion and should be considered as a focal point of future research in this area.

**Figure 10:**
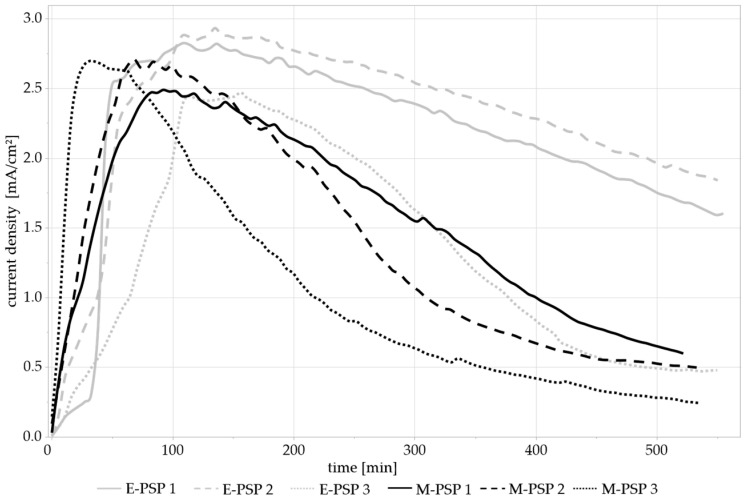
The PSP of the bending test specimens over 9.25 h. The PSPs of the electrochemical control group (E-PSP 1–3) are compared with the PSPs under additional mechanical stress (M-PSP 1–3).

Applying a static potential was intended to simulate an accelerated corrosion process (as may be caused by a more corrosive medium or the formation of a galvanic element) to provoke an effect on the mechanical integrity of the samples. However, no such effect could be observed. Figure 10 shows the current density during PSP at −0.95 V_Ag/AgCl_ without (E-PSP 1–3) and with (M-PSP 1–3) concurrent dynamic mechanical stress. All six PSP curves show a comparable course, with the current density rising quickly to a maximum and then gradually decreasing. It is noticeable that two of the measurements without mechanical loading showed a steeper decline during the course of the measurement than the other samples.

The results show that the impact of mechanical loading on the electrochemical processes acting on the samples in SBF can be measured in the presented experimental setup. This allows for an effective investigation of the corrosion fatigue properties of different metallic materials for bioresorbable implants. Furthermore, it could be shown that the monitoring of the current and, therefore, the corrosion reaction rate, is a feasible possibility for the investigation of corrosion fatigue properties.

In future experiments, different electrochemical techniques can be utilized in conjunction with the presented setup to investigate specific material characteristics. Similarly to the techniques outlined by Talbot et al. [21,34], current transients caused by the formation of cracks, pitting, or other surface defects could be detected by statically polarizing a sample at the corrosion potential. By means of other electrochemical methods, like potentiodynamic polarization or electrochemical impedance spectroscopy (EIS), before, during, and/or after fatigue testing, the effects of mechanical loading on corrosion processes could be investigated further [19,35].

### 3.4. SEM Images

**Figure 11:**
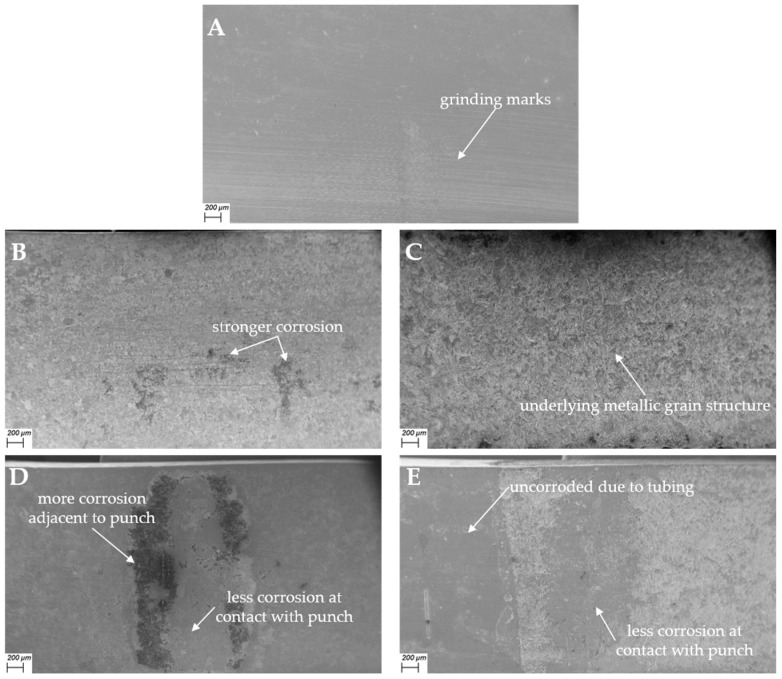
SEM images with most important findings of one used sample per group at 60× magnification: (**A**) Air1, (**B**) E-OCP1, (**C**) E-PSP1, (**D**) M-OCP1, and (**E**) M-PSP3.

Figure 11 shows SEM images of the corrosion fatigue groups M-OCP and M-PSP, as well as the control groups Air, E-OCP, and E-PSP in 60× magnification. The images of the E-OCP and E-PSP samples show the surface which was exposed to the medium. The images of the M-OCP and M-PSP samples show the areas where the punch pushed onto the surface during fatigue testing, as well as the adjacent surface areas that were exposed to the medium. On the SEM image of the sample measured in air (Figure 11A), grinding marks are visible. The E-OCP sample (Figure 11B) shows uniform surface corrosion, with some areas indicating slightly stronger signs of corrosion. Some marks from grinding are still visible. The E-PSP sample (Figure 11C) shows no recognizable grinding marks but exhibits a uniformly and more severely corroded surface, potentially revealing the underlying metallic grain structure. The M-OCP surface (Figure 11D) is slightly corroded in general as well as in the center of the contact area between the punch and the sample. The area directly adjacent to the contact area is more severely corroded. Figure 11E shows that the area of the M-PSP sample that was protected by the tubing (on the left) is uncorroded. In contrast, the exposed area of the sample is strongly corroded. However, the contact area of the sample and support pin (middle) is noticeably less corroded.

Generally, the areas on the samples’ surfaces that were in contact with the support and punch exhibit minimal corrosion. Consequently, the mechanically loaded samples may have a smaller overall surface area contributing to the current flow. This effect could account for the lower measured current density. This should be kept in mind when using techniques involving current measurement as a part of the investigation of corrosion fatigue behavior, as they should be viewed and interpreted mainly as qualitative rather than quantitative methods.

## 4. Conclusions

The presented experimental setup lays the groundwork for a more extensive investigation of the corrosion fatigue characteristics of biodegradable implant materials. The improvements in both the setup and methodology hold significant promise for furthering the overall understanding of how corrosion and material fatigue interact. The investigation of biodegradability in vitro poses several challenges, and the presented study is only the foundation. The following will outline several possibilities for future research.

Especially in combination with long-term immersion experiments, the presented methodology shows great potential for furthering our understanding of the effect that corrosion processes have on the fatigue characteristics of biodegradable implant materials and vice versa. In future experiments, samples could be immersed and stored in corrosive media for pre-defined durations prior to corrosion fatigue experiments to investigate the fatigue behavior of samples at different stages of degradation. Furthermore, the maximum number of loading cycles should be increased to better investigate the effects of a corrosive medium on the fatigue characteristics. Different metals and alloys can be used, and the parameters can be adjusted accordingly by applying the outlined methodology, as well as a variety of aqueous media, to simulate a range of environmental conditions. By using polarization techniques and applying different potentials, the passivation characteristics, pitting and passive layer breakdown during mechanical loading, and crack initiation can be effectively investigated in the future. By polarizing the samples at the corrosion potential, current transients caused by the formation of cracks or surface defects may be able to be detected.

This work provides a reproducible setup and experimental framework that can be implemented universally, facilitating continued research and enabling further advancements in the field.

## Figures and Tables

**Table 1:** Ion concentration in DPBS^− −^ used as SBF.

Ion	Ion Concentration in DPBS^− −^ [mM]
${Na}^{+}$	146.00
$K^{+}$	4.18
${Mg}^{2+}$	-
${Ca}^{2+}$	-
${Cl}^{-}$	139.50
${SO}_{4}^{2-}$	-
${HPO}_{4}^{2-}$	9.56

**Table 2:** Maximum deflection and evaluation of termination criterion (1) sample failure due to fracture or reaching maximum deflection of −1 mm, or (2) reaching of predefined maximum number of 5 × 10^5^ loading cycles.

Applied Force and Sample (a) or (b)	Maximum Deflection [mm]	Evaluation Termination Criterion
20 N	−3.26	(1) → failure
15 N	−2.31	(1) → failure
10 N (a)	−0.6	(2) → success
5 N (a)	−0.03	(2) → success
8.25 N (a)	−0.05	(2) → success
10 N (b)	−0.14	(2) → success
8.25 (b)	−0.06	(2) → success
5 N (b)	−0.023	(2) → success

**Table 3:** Relative deviation between actual force and set force in dynamic three-point bending test.

Set Force [N]	Relative Deviation from Set Force [%]
5.00	3.96 ± 0.01
8.25	1.80 ± 0.03
10.00	4.20 ± 1.10

## Data Availability

The datasets presented in this article are not readily available due to technical limitations. Requests to access the datasets should be directed to the corresponding author.
